# Reinfection rates after one- and two-stage revision surgery for hip and knee arthroplasty: a systematic review and meta-analysis

**DOI:** 10.1007/s00402-021-04190-7

**Published:** 2021-09-30

**Authors:** Annemarie L. Goud, Netanja I. Harlianto, Solaiman Ezzafzafi, Ewout S. Veltman, Joris E. J. Bekkers, Bart C. H. van der Wal

**Affiliations:** 1 Department of Orthopedics, Clinical Orthopedic Research Center midden-Nederland (CORC-mN), Diakonessenhuis Hospital Utrecht/Zeist, PO Box 80250, 3508 TG Utrecht, The Netherlands; 2 Department of Orthopedics, University Medical Center Utrecht, Heidelberglaan 100, 3508 GA Utrecht, The Netherlands

**Keywords:** Periprosthetic joint infection, Revision arthroplasty, Arthroplasty, Reinfection, One-stage, Two-stage

## Abstract

**Purpose:**

Revisions for periprosthetic joint infection of knee and hip arthroplasty can be performed following one- or two-stage treatment protocols. Current literature is inconclusive whether one protocol is superior to the other, as prior literature reported similar reinfection rates for both treatment options. We aimed to provide a systematic review and meta-analysis of current literature on septic arthroplasty revisions.

**Methods:**

Between April 2015 and December 2020, Medline, Embase, and The Cochrane Library were searched for studies reporting reinfection outcomes in patients treated with one-stage and two-stage knee or hip revision arthroplasty. Two reviewers independently extracted data and disagreements were resolved by a third investigator. We utilized a double arcsine transformation, prior to pooling using a random-effects model.

**Results:**

For hip revision arthroplasty, we identified 14 one-stage studies (*n* = 1237) with a pooled reinfection rate of 5.7% (95% CI 3.7–8.1%), and 46 two-stage studies (*n* = 5009) with a reinfection rate of 8.4% (95% CI 6.9–9.9%). For knee revision arthroplasty, 6 one-stage studies (*n* = 527) and 48 two-stage studies (*n* = 4344) were identified with reinfection rates of 12.7% (7.0–19.7%) and 16.2% (13.7–19.0%), respectively. Overall, reinfection rates did not vary substantially after subgroup analysis. Limitations of our study are the limited amount of one-stage studies that introduce a potential bias.

**Conclusion:**

The reinfection rates following one- and two-stage hip and knee arthroplasty revisions were similar. Knee reinfection rates have increased compared to the previous analysis. Individual patient characteristics and adequate treatment algorithms are needed for a more individual selection approach, until a randomized trial is performed.

**Supplementary Information:**

The online version contains supplementary material available at 10.1007/s00402-021-04190-7.

## Introduction

Total hip and knee arthroplasties have been steadily increasing over the years and are expected to increase even further the coming decades. A well-known complication following total joint arthroplasty is periprosthetic joint infection (PJI), with reported incidence rates around 2%. Acute PJI is treated with debridement, antibiotics, and implant retention (DAIR), usually within 2–4 weeks of onset. Local and systemic antibiotics, combined with the debridement of necrotic and infected tissue aims to eliminate infection, prevent infection relapse, and restore function [1–3]. If, however, the DAIR is not successful, a one-stage or two-stage revision procedure is the logical next treatment step. During a one-stage procedure, the infected prosthesis is removed followed by radical debridement of infected tissue and direct re-implantation of a new prosthesis. Alternatively, a two-stage procedure can be used with a time interval between the removal and the re-implantation of the prosthesis, during which antibiotics are given depending on the cultured pathogen, with or without a temporary spacer. Two-stage revision makes it possible to evaluate response to antibiotic therapy, perform a second debridement if needed, and better predict treatment success [4, 5]. However, two-stage revision is more expensive, can potentially increase morbidity, and patients are hospitalized for a longer period [5, 6]. Furthermore, patients are impaired from their normal daily activities between surgery intervals, and the interim spacer or temporary implant can also introduce more complications such as spacer luxation [7].

Previously, different studies have been performed investigating the reinfection rates following revision arthroplasty. Similar reinfection rates have been reported for both one-stage and two-stage revision arthroplasty for the hip and knee. Beswick et al. [8] reported reinfection rates of 8.6% (95% CI = 4.5–13.9%) for one-stage hip revision arthroplasty and 10.2% (95% CI = 7.7–12.9%) for two-stage hip revision. Also, Lange et al. [9] reported reinfection rates of 13.1% and 10.4% for one-stage and two-stage hip revision arthroplasty, respectively.

The most recent published meta-analysis by Kunutsor et al. [10] on hip revision arthroplasty reported reinfection rates for one-stage revision at 8.2% (6–10.8%) and 7.9% (6.2–9.7%) for two-stage revision. For knee revision arthroplasty, Kunutsor et al. [11] reported reinfection rates for one-stage and two-stage revision of 7.6% (3.4–13.1%) and 8.8% (7.2–10.6%), respectively. Since the latest meta-analyses in 2015, the number of studies reporting reinfection rates following knee and hip revision arthroplasty has increased. As previous meta-analyses reported similar reinfection rates, and were inconclusive as to which protocol is superior to the other, we aimed to provide a summary of studies that have been published in the last 5 years reporting reinfection rates in one-stage and two-stage revision surgery for knee arthroplasty and hip arthroplasty.

## Methods

### Data sources and searches

This systematic review and meta-analysis was carried out in accordance with the Preferred Reporting Items for Systematic Reviews and Meta-Analysis (PRISMA) statement [12]. A systematic literature search of Medline, Embase, and Cochrane was performed for articles published between April 2015 and December 2020 using a combination and variation of the terms “reinfection”, “one-stage”, “two-stage”, “knee”, “hip”, and “revision arthroplasty”. For each database, a specific search was generated and converted accordingly. No language restrictions were applied. The full search can be found in Supplementary material, Appendix A. No institutional review board approval was required for the current study.

### Study selection

We included studies that reported reinfection rates in patients undergoing one-stage or two-stage revision for hip or knee arthroplasty. Studies with selected patient groups, with a follow-up of less than 2 years, and with less than ten participants were excluded. Also, systematic reviews and ongoing trials were excluded. Two independent reviewers (N.I.H. and S.E) selected relevant studies, and consensus was resolved by a third investigator (E.S.V.). Potential overlapping studies included in previous reviews were identified and excluded.

### Data extraction and critical appraisal

N.I.H. independently extracted data, which was subsequently compared by S.E. to the original citation. Extracted data from eligible studies included: first author, publication year, country, inclusion period, number of reinfections, study size, mean age, percentage of males, and follow-up after re-implantation. To assess the quality of each study, we utilized the Methodological Index for Non-Randomized Studies (MINORS) criteria [13], which assigns a score to each study by taking into account a stated aim, consecutive patients, prospective data collection, appropriate end point, unbiased assessment of the end point, appropriate follow-up period, less than 5% loss of follow-up, a prospective calculation of study size, adequate control group, baseline equivalence of groups, and adequate statistical analysis. Each item is assigned a total of two points. The maximum score for non-comparative studies is 16, and 22 for comparative studies. N.I.H and S.E independently scored each study, and discussed reported differences.

### Data analysis

The primary outcome (reinfection rate) was reported with 95% confidence intervals (95% CI) and analyzed using a random-effects model. To quantify heterogeneity, we utilized Higgin’s & Tompson’s *I*^2^ statistic. Low heterogeneity was defined as *I*^2^ < 50%, moderate heterogeneity as *I*^2^ = 50–75%, and high heterogeneity as *I*^2^ > 75% [14]. Following this, we performed a random effect meta-regression and stratified analysis to further investigate heterogeneity of the outcome using study location, study size, quality score, age, and year of publication. To stabilize the variance of raw proportions we performed a Freeman–Tukey double arcsine square root transformation [15]. The Wilson score interval method was used for confidence intervals [16]. Publication bias was evaluated using Egger’s regression symmetry test [17]. If there was evidence of publication bias, we used the trim and fill method from Duval and Tweedie to evaluate the imputation of the “unavailable” studies to adjust for publication bias [18]. Data analysis was done using R version 3.6.3 (R Foundation for Statistical Computing, Vienna, Austria), using the meta and metafor package [19, 20].

## Results

**Fig. 1:**
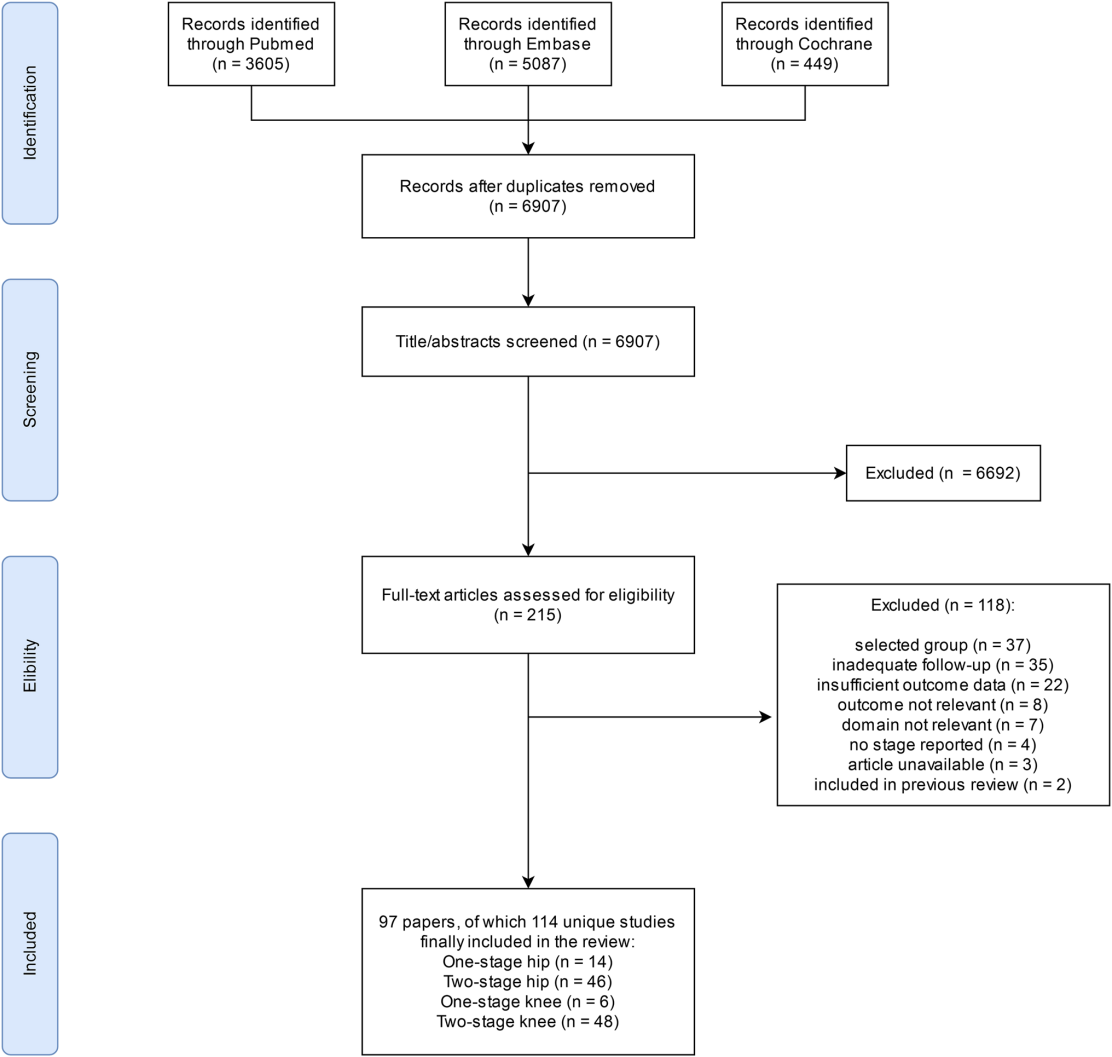
PRISMA flowchart of study selection

A total of 6907 citations were identified after the removal of duplicates. Following title and abstract screening, 215 citations were assessed for full text eligibility. Of these, 118 studies were excluded which resulted in 97 studies included in the final analysis (Fig. 1). A total of 6246 hip revisions with 557 reinfections and 4871 knee revisions with 863 reinfections were included in the meta-analysis.

### Study characteristics and quality

Study characteristics are summarized in Table 1 for both hip and knee revision arthroplasty. The median age for knee revision was 69 and 68.2 years for one- and two-stage, respectively. The median percentage of males was around 46%. For hip revision, the mean ages were 66.5 and 65.3 years for one- and two-stage, respectively. A higher percentage was male (59% and 54.3%, respectively). A more detailed description of each study is available in Supplementary material, Appendix B. No published results of clinical trials were identified comparing one-stage and two-stage for hip or knee revision arthroplasty. We only identified retrospective and prospective longitudinal (cohort) studies. The range of the reported MINORS scores was 11–16, with most studies scoring either 12 (34.8%) or 13 (25.2%).

**Table 1:** Summary of population characteristics for both knee and hip revision arthroplasty

	Knee		Hip	
	One-stage	Two-stage	One-stage	Two-stage
No. of studies	6	48	14	46
Total number of participants	527	4344	1237	5009
Total number of reinfections	78	785	85	472
Mean age in years, median (IQR)	69 (67.6–70)	68.2 (66.1–70)	66.5 (60.5–69.3)	65.3 (63.2–68)
Percentage male, median (IQR)	46.6 (43.7–48.8)	46.3 (37–52.1)	59 (54–61.5)	54.3 (49–57.8)
Mean follow-up in months, median (IQR)	52.9 (44–61.2)	52 (39.7–71.1)	66 (58–102.8)	57.2 (46–68.5)

*IQR* interquartile range

### One-stage hip revision

**Fig. 2:**
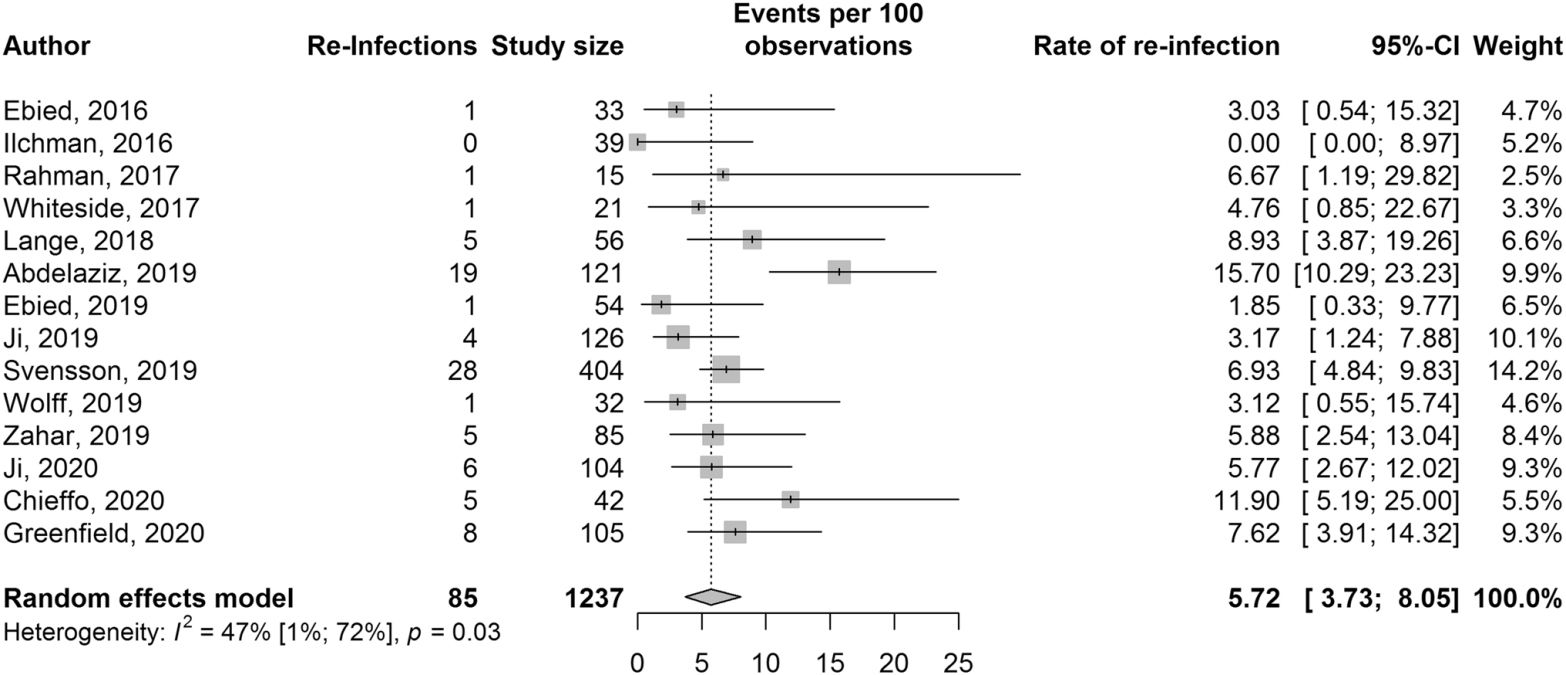
Forest plot for one-stage hip revision reinfection rates presented with 95% confidence intervals (95% CI)

Fourteen studies consisting of a total of 1237 pooled participants with 85 reported reinfections for one-stage hip revision were identified (Fig. 2). The random-effects pooled reinfection rate was 5.7% (95% CI 3.7–8.1%). There was evidence of low heterogeneity, *I*^2^ = 47% (95% CI 1–72%, *p* = 0.03). After exploration with meta-regression and subgroup analysis, no heterogeneity could be further explained (Supplementary material, Appendix C). Egger’s regression test was not significant (*p* = 0.58), which indicates no evidence of publication bias.

### Two-stage hip revision

**Fig. 3:**
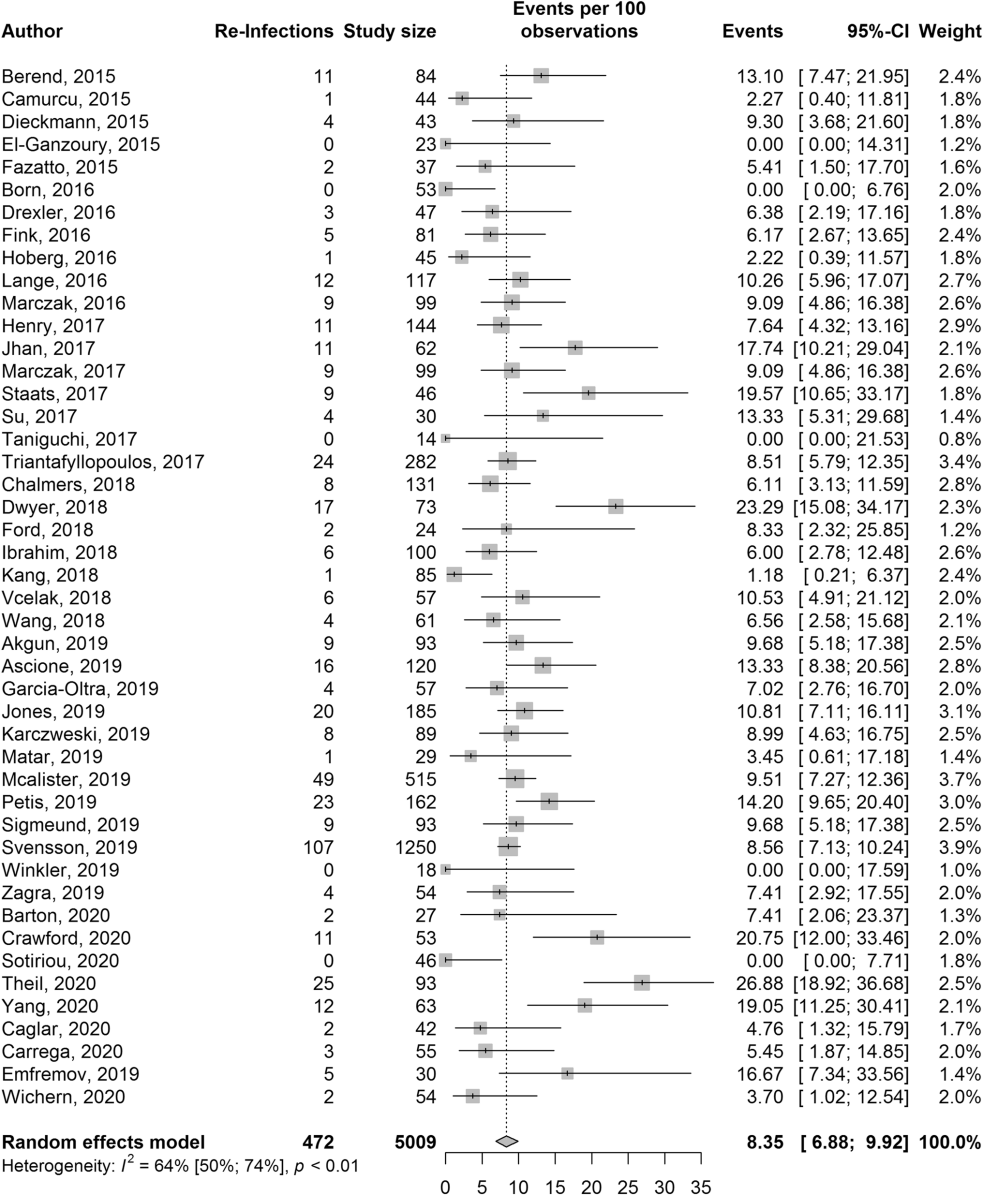
Forest plot for two-stage hip revision reinfection rates presented with 95% confidence intervals (95% CI)

Forty-six studies for two-stage hip revision consisting of 5009 pooled participants with 472 reported reinfections were identified (Fig. 3). The pooled random-effects reinfection rate was 8.4% (95% CI 6.9–9.9%). There was evidence of moderate heterogeneity, *I*^2^ = 64% (95% CI 50–74%, *p* < 0.01), which was not explained after exploration of heterogeneity (Supplementary material, Appendix C). Egger’s regression test for publication bias was not significant (*p* = 0.62).

### One-stage knee revision

**Fig. 4:**
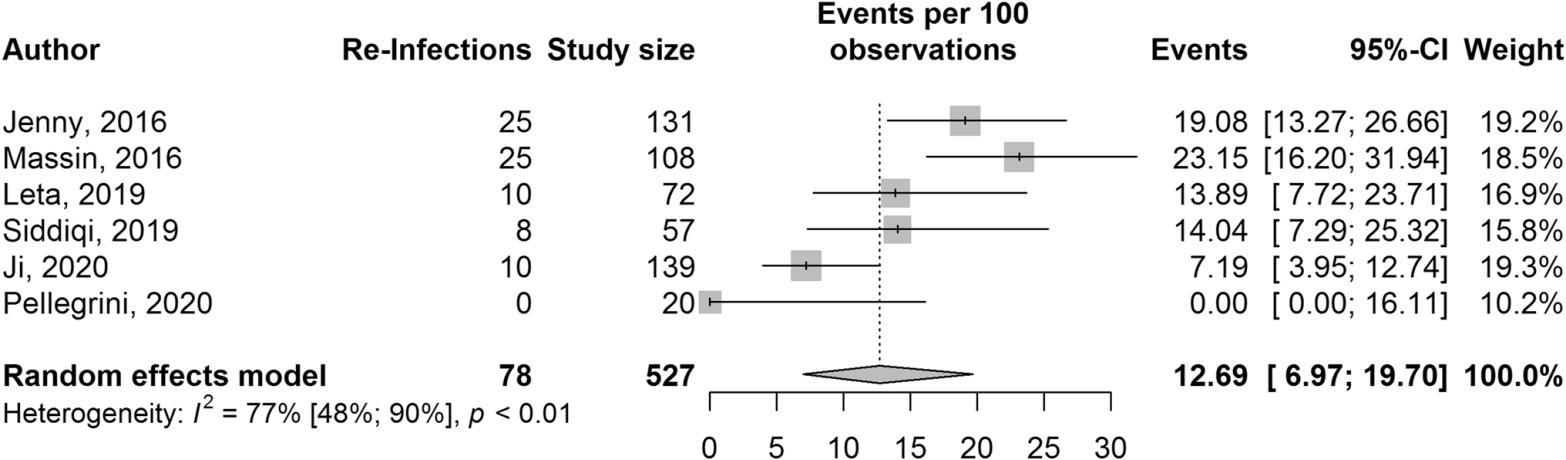
Forest plot for one-stage knee revision reinfection rates presented with 95% confidence intervals (95% CI)

Reinfection outcomes were reported in six studies with a total of 527 pooled participants and 78 reinfections (Fig. 4). The pooled reinfection rate (95% CI) was 12.7% (7.0–19.7%). *I*^2^ = 77% (95% CI 48–90%, *p* < 0.01). Subgroup analysis and meta-regression could not further explain heterogeneity (Supplementary material, Appendix C). As the number of studies was less than 10, Egger’s regression test was not performed.

### Two-stage knee revision

**Fig. 5:**
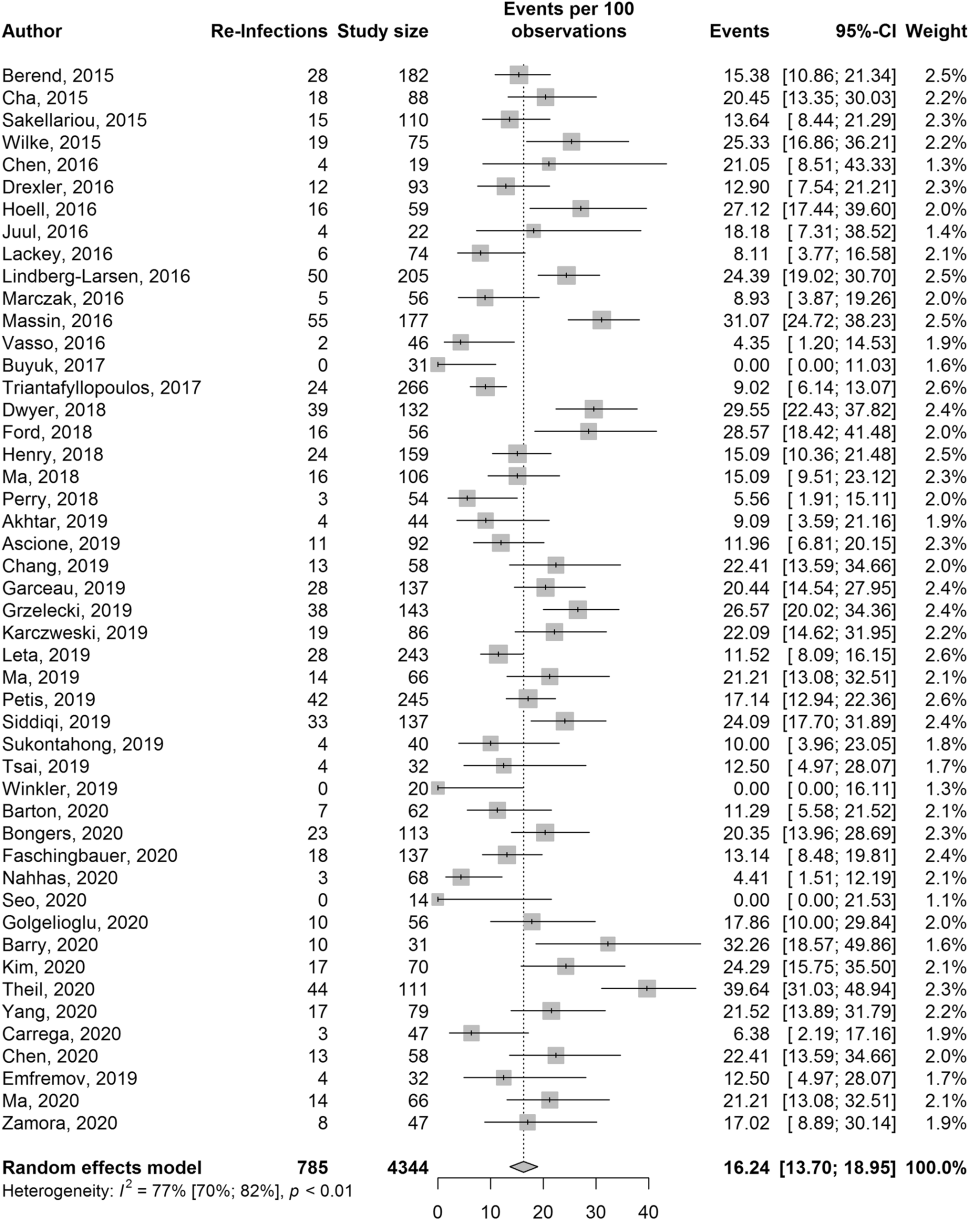
Forest plot for two-stage knee revision reinfection rates presented with 95% confidence intervals (95% CI)

Reinfection outcomes were reported in 48 studies with a total of 4344 pooled participants and 785 reinfections (Fig. 5). The pooled reinfection rate (95% CI) was 16.2% (13.7–19.0%). Heterogeneity was high, (*I*^2^ = 77%, 95% CI 70–82%, *p* < 0.01). Heterogeneity could not be further explained following subgroup analysis and meta-regression (Supplementary material, Appendix C). There was no evidence of publication bias: Egger’s test: *p* = 0.21.

## Discussion

### Main findings

We aimed to assess reinfection rates in both one- and two-stage revision for PJI in both knee and hip revision arthroplasty. Our study reports the most recent evidence of reinfection rates for one- and two-stage knee and hip revision arthroplasty using studies published in the last 5 years. The pooled reinfection rates for hip revision arthroplasty were 5.7% (95% CI 3.7–8.1%), and 8.2% (95% CI 6.8–9.8%) for one- and two-stage, respectively. For knee revision arthroplasty, the reinfection rate for one-stage was 12.7% (7.0–19.7%) and for two-stage revision, this was 16.2% (13.7–19.0%). The pooled reinfection rates were characterized by low to high heterogeneity. Moreover, we analyzed and explored heterogeneity using study population characteristics, in which the overall reinfection rates remained the same after meta-regression and subgroup analysis. In line with previous meta-analyses, similar reinfection rates were found in our study between one- and two-stage revision for both knee [11] and hip [8–10] revision arthroplasty. The reinfection rates in our study for both one- and two-stage knee revision arthroplasty, however, were surprisingly higher when compared to the previous meta-analysis [11]. Possible explanations for this observed increase are older studies underreporting reinfection rates, or the improved diagnostic management for PJI in recent decades. The risk for reinfection is influenced by surgical technique and infection management strategies over time. In general, studies with a larger patient population reported higher reinfection rates for knee arthroplasty in our results.

Comparing factors and outcomes in one- and two-stage procedures for PJI can be quite arduous as the outcomes can be influenced by many variables. Increased reinfection rates have been reported to be associated with the same organism as the previous infection [21], while other authors have associated multi-organism infections or resistance to antibiotics with higher reinfection rates in knee revision arthroplasty [22].

It remains challenging to identify patient characteristics as a basis for one- or two-stage treatment selection in patients with PJI. For chronic PJI, a one-stage protocol is favored for subjects in which difficult to treat microorganisms (DTT) are less probable, and for subjects with intact bone and soft tissue without a previous history of revisions. Thus, a two-stage protocol is preferred for patients with DTT pathogens, bad bone or soft tissue, fistula, or a history of multiple previous revisions. The extent of the period of antibiotic treatment is influenced by the DTT pathogen and the condition of bone/soft tissue [23].

We performed subgroup analysis to a certain extent, but a comprehensive analysis of patient characteristics, such as antibiotic schedule or the use of spacers, was limited due to the lack of available data. In a meta-analysis of pooled longitudinal studies male sex, smoking status, diabetes, rheumatoid arthritis, and a history of joint surgery were identified as patient-related risk factors for PJI [24]. The largest cohort study to date, identified male sex, age, elevated body mass index (BMI), and American Society of Anesthesiologist (ASA) grade as risk factors for PJI [25]. Citak et al. [26] identified weight > 100 kg, polymicrobial infections, persistent wound drainage, wound revision, the isolation of streptococcus and enterococcus species as risk factors for reinfection in one-stage knee revision arthroplasty. Specifically for reinfection in two-stage knee revision arthroplasty, inflammatory arthritis, hematoma formation, and staphylococcus carriers were identified as risk factors for reinfection [27]. For two-stage hip revision arthroplasty, Jhan et al.[28] identified BMI > 30 kg/m^2^, liver cirrhosis, gram-negative bacteria, and concurrent sinus tract infections as independent risk factors for reinfection. And finally, in a study using one-stage hip arthroplasty patients reviewed for PJI, prolonged wound drainage, and previous septic revision were identified as independent risk factors for reinfection [29]. Using individual patient characteristics and adequate treatment algorithms, a more individual approach for the selection of patients for either one- or two-stage revision can be achieved. Recently, Kilgus et al. [30] studied patient independent factors for infection persistence following failed septic revisions. In 85% of failed cases, patient independent factors could be identified, in which failure was most frequently caused by inadequate treatment algorithms. In their patient cohort, high rates of infection eradication were achieved following extensive and critical review of previous treatment using a checklist algorithm.

For our analysis, only retrospective and prospective observational studies were included. The studies in our analysis can be subject to selection bias, due to the heterogeneous patient selection criteria within each study, surgeon preferences, and different hospital protocols for the allocation of patients to either one- or two-stage revision. These confounders are mostly unknown, but can influence our pooled results, as it is possible that patients with a greater disease burden (e.g. resistant microorganisms) are more likely to be offered a two-stage approach. Although some studies reported similar reinfection outcomes between “difficult to treat” and “easy to treat” microorganisms, this aspect remains inconclusive to date [31]. While two-stage revision is more frequently performed, one-stage revision is done in select centers and hospitals. Better functional outcomes have been reported for both one-stage knee and hip revision compared to two-stage revision, including post-operative patient reported outcome measures [32–34]. Guidelines exist for using one-stage approach with a select set of criteria and contraindications. Different protocols have been established, such as the one-stage criteria of the Infectious Diseases Society of America (IDSA), or at the International consensus meeting [35]. For one-stage knee-revision, the usage of strict patient selection offered good results with low reinfection rates, but comparable results were also achieved in studies not adhering to strict protocols [36].

Although no published clinical trials comparing one-stage and two-stage were found in the literature, a published protocol for the INFection ORthopaedic Management (INFORM) trial was identified, which is currently ongoing, and aims to sort out the reinfection rates between one-stage and two-stage hip revision in a randomized trial [37]. Specifically for reinfection in revision arthroplasty, additional evaluation of large pooled cohorts and prospective comparative studies are needed for the identification of individual patient and microbiology characteristics, which could lead to more patient specific treatment algorithms.

### Strengths and limitations

A strength of our study is the inclusion of the most recent available studies reporting reinfection rates in revision arthroplasty with the inclusion of a large patient group for both hip and knee revision arthroplasty. We also assessed publication bias, and explored heterogeneity and study-specific bias. The main limitation of our study is the pooling of observational studies, which introduces selection bias. Secondly, the analyses for knee revision arthroplasty were characterized by high heterogeneity. Furthermore, the small amount of one-stage studies available for both knee and hip revision arthroplasty could have introduced bias, making the comparison between one- and two-stage less reliable. Taking into account the lack of published one-stage studies, restricted data, and high heterogeneity, care should be taken when construing our results.

## Conclusion

The reinfection rates between one- and two-stage were similar, with knee revision arthroplasty having higher reinfection rates compared to the previous meta-analysis. Individual patient characteristics and adequate treatment algorithms are needed for a more individual selection approach, until a randomized trial is performed.

## Supplementary Information

Below is the link to the electronic supplementary material.

Supplementary file1 (DOCX 811 kb)

## Data Availability

The authors confirm that the data supporting the findings of this study are available within the article and its supplementary materials.

## Code availability

Available upon reasonable request.
