# Supplementary material for: Reinfection rates after one- and two-stage revision surgery for hip and knee arthroplasty: a systematic review and meta-analysis
Source: Arch Orthop Trauma Surg. 2021 Sep 30;143(2):829–38. doi: 10.1007/s00402-021-04190-7 (PMC9925475; doi:10.1007/s00402-021-04190-7)
Supplement: Supplementary file 1 — Supplementary file1 (DOCX 811 kb) [file 402_2021_4190_MOESM1_ESM.docx]

**Supplementary material**

| **Appendix A** | Search strategy |
| --- | --- |
| **Appendix B** | Study characteristics |
| **Appendix C** | Subgroup analysis and Meta regression |
| **Figure 1** | One-stage hip revision |
| **Figure 2** | Two-stage hip revision |
| **Figure 3** | One-stage knee revision |
| **Figure 4** | Two-stage knee revision |

**Appendix A. Systematic search strategy.**

We queried studies published between April 2015 and December 2020 in Medline, Embase, and the Cochrane library. No language restrictions were applied. Each search was transformed according to each specific database.

**PUBMED SEARCH**

**1.1 Domain: infection**Infection[MeSH] OR Infecti*[tiab] OR Reinfection[tiab] OR Re-infection[tiab] OR Prosthesis Infection[tiab] OR Prosthesis-Related Infections[tiab] OR PJI[tiab] OR Periprosthetic Joint Infection[tiab] OR Peri-prosthetic Joint Infection[tiab] OR Periprosthetic Knee Infection[tiab] OR

Periprosthetic Hip Infection[tiab] OR Implant infection[tiab] OR Implant related infection[tiab] OR Arthoplasty Infection[tiab] OR Sepsis[MeSH] OR Sepsis[tiab]

**AND**

**2.1 Determinant: Arthroplasty**

Arthroplasty, Replacement[MeSH] OR Joint Prosthesis[MeSH] OR Knee Prosthesis[tiab] OR Hip Prosthesis[tiab] OR Arthroplasty[tiab] OR Hip Replacement[tiab] OR Knee Replacement[tiab]

**AND**

**2.2 Determinant: Operation**

1-stage[tiab] OR 2-stage[tiab] OR one-stage[tiab] OR two-stage[tiab] OR single stage[tiab] OR single-stage[tiab] OR Reoperation[MeSH] OR Reoperation[tiab] OR Revision Surg*[tiab] OR Joint Revision[tiab] OR Repeat Surgery[tiab] OR Replantation[MeSH] OR Reimplant*[tiab] OR Staged Revision[tiab] OR Revision Arthroplasty[tiab] OR Replacement[tiab] OR exchange[tiab]

**(1.1 Domain: infection AND 2.1 Determinant: Arthroplasty AND 2.2 Determinant: Operation)**

**EMBASE SEARCH**

- 1. **Domain: infection**

'infection'/exp OR ‘infecti*’:ti,ab,kw OR ‘Reinfection’:ti,ab,kw OR 'sepsis'/exp OR ‘Sepsis’:ti,ab,kw

**AND**

**2.1 Determinant: Arthroplasty**

'arthroplasty'/exp OR 'hip prosthesis'/exp OR 'knee prosthesis'/exp OR ‘Knee Prosthesis’:ti,ab,kw OR ‘Hip Prosthesis’:ti,ab,kw OR ‘Arthroplasty’:ti,ab,kw OR ‘Hip Replacement’:ti,ab,kw OR ‘Knee Replacement’:ti,ab,kw

**AND**

**2.2 Determinant: Operation**

'reoperation'/exp OR 'reimplantation'/exp OR ‘1-stage’:ti,ab,kw OR ‘2-stage’:ti,ab,kw OR ‘one-stage’:ti,ab,kw OR ‘two-stage’:ti,ab,kw OR ‘single-stage’:ti,ab,kw OR ‘Reoperation’:ti,ab,kw OR ‘Revision Surg*’:ti,ab,kw OR ‘Joint Revision’:ti,ab,kw OR ‘Repeat Surgery’:ti,ab,kw OR ‘Reimplant*’:ti,ab,kw OR ‘Staged Revision’:ti,ab,kw OR ‘Revision Arthroplasty’:ti,ab,kw OR ‘Replacement’:ti,ab,kw OR ‘exchange’:ti,ab,kw

**(1.1 Domain: infection AND 2.1 Determinant: Arthroplasty AND 2.2 Determinant: Operation)**

**COCHRANE SEARCH**

**1.1 Domain: infection**MeSH descriptor: [Infections] explode all trees OR (infecti*):ti,ab,kw OR Reinfection:ti,ab,kw OR PJI:ti,ab,kw

**AND**

**2.1 Determinant: Arthroplasty**

MeSH descriptor: [Arthroplasty] explode all trees OR MeSH descriptor: [Joint Prosthesis] explode all trees OR Knee Prosthesis:ti,ab,kw OR Hip Prosthesis:ti,ab,kw OR Arthroplasty:ti,ab,kw OR Hip Replacement:ti,ab,kw OR Knee Replacement:ti,ab,kw

**AND**

**2.2 Determinant: Operation**

MeSH descriptor: [Reoperation] explode all trees OR MeSH descriptor: [Replantation] explode all trees OR one-stage:ti,ab,kw OR two-stage:ti,ab,kw OR single stage:ti,ab,kw OR Reoperation:ti,ab,kw OR Revision Surg*:ti,ab,kw OR Joint Revision:ti,ab,kw OR Repeat Surgery:ti,ab,kw OR Reimplant*:ti,ab,kw OR Staged Revision:ti,ab,kw OR Revision Arthroplasty:ti,ab,kw OR Replacement:ti,ab,kw OR Exchange:ti,ab,kw

**(1.1 Domain: infection AND 2.1 Determinant: Arthroplasty AND 2.2 Determinant: Operation)**

**Appendix B.**

Table B.1: Summary characteristics for hip revision arthroplasty;
Study characteristics of hip revision arthroplasty are displayed using the number of patients, number of reinfections, the mean age in years, mean follow-up in months, and the percentage of males in each study. N: number of patients F/U: follow-up; NS: not specified;

| Study | Study Period | Age in years | %Male | F/U in months | Stage | N | Reinfection | Minors |
| --- | --- | --- | --- | --- | --- | --- | --- | --- |
| Ebied, 2016 | 2006-2011 | 61 | 60.1 | 72 | One | 33 | 1 | 14 |
| Ilchman, 2016 | 1996-2011 | NS | 51.3 | 79.2 | One | 39 | 0 | 13 |
| Rahman, 2017 | 1997-2012 | 58.9 | 40 | 102.8 | One | 15 | 1 | 12 |
| Whiteside, 2017 | 2002-2013 | 67 | 71.4 | 63 | One | 21 | 1 | 14 |
| Lange, 2018 | 2009-2014 | 72 | 55 | 48 | One | 56 | 5 | 15 |
| Abdelhaziz, 2019 | 2009-2017 | 67.5 | 55 | 66 | One | 121 | 19 | 13 |
| Ebied, 2019 | 2009-2014 | NS | NS | 60 | One | 54 | 1 | 15 |
| Ji, 2019 | 2010-2016 | 58.7 | 54 | 58 | One | 126 | 4 | 14 |
| Svensson, 2019 | 1979-2015 | 70 | 60.9 | 130.8 | One | 404 | 28 | 12 |
| Wolf, 2019 | 1993-2008 | 36.8 | 61.5 | 180 | One | 32 | 1 | 11 |
| Zahar, 2019 | 2006-2007 | 66 | 63.5 | 126 | One | 85 | 5 | 12 |
| Chieffo, 2020 | 2010-2015 | 69 | 62 | 32 | One | 42 | 5 | 12 |
| Greenfield, 2020 | 2006-2015 | 70.1 | 59 | 51.6 | One | 105 | 8 | 13 |
| Ji, 2020 | 2009-2016 | 62.7 | 43.2 | > 24 | One | 104 | 6 | 13 |
| Berend, 2015 | 2006-2013 | 62 | 52 | 36 | Two | 84 | 11 | 12 |
| Camurcu, 2015 | 2005-2011 | 62 | 56 | 54 | Two | 44 | 1 | 12 |
| Dieckmann, 2015 | 2000-2020 | 66 | 49 | 46.3 | Two | 43 | 4 | 12 |
| El-Ganzoury, 2015 | 2006-2015 | 45 | NS | 48 | Two | 23 | 0 | 14 |
| Fazatto, 2015 | 1999-2013 | NS | NS | 95 | Two | 37 | 2 | 12 |
| Born, 2016 ­ | 1993-2012 | NS | NS | 60 | Two | 53 | 0 | 12 |
| Drexler, 2016 | 2010-2013 | 67 | 49 | 31 | Two | 47 | 3 | 12 |
| Fink, 2016 | 2004-2016 | 70.7 | 53.3 | 51.2 | Two | 81 | 5 | 14 |
| Hoberg, 2016 | 2004-2008 | NS | NS | 55 | Two | 45 | 1 | 11 |
| Lange, 2016 | 2003-2008 | 68 | 57 | 94.8 | Two | 117 | 12 | 13 |
| Marczak, 2016 | 2006-2014 | 59.4 | NS | 52.2 | Two | 99 | 9 | 12 |
| Henry, 2017 | 2009-2014 | 65 | 40 | >24 | Two | 144 | 11 | 12 |
| Jhan, 2017 | 2005-2012 | 57 | 69.3 | 68.4 | Two | 62 | 11 | 12 |
| Marczak, 2017 | 2006-2014 | 63.3 | 60.2 | 52.2 | Two | 99 | 9 | 12 |
| Staats, 2017 | 2001-2014 | 64.3 | 54.3 | 46 | Two | 46 | 9 | 13 |
| Su, 2017 | 2001-2012 | 47.3 | 60 | 36 | Two | 30 | 4 | 12 |
| Taniguchi, 2017 | 2008-2012 | 69.4 | 57.1 | 58.8 | Two | 14 | 0 | 13 |
| Triantafyllopoulos, 2017 | 1998-2014 | 65.4 | 51.6 | 132.2 | Two | 282 | 24 | 13 |
| Chalmers, 2018 | 2005-2013 | 65 | 58 | 60 | Two | 131 | 8 | 14 |
| Dwyer, 2018 | 2000-2014 | 63.4 | 51.7 | 62.4 | Two | 73 | 17 | 12 |
| Ford, 2018 | 2004-2014 | 61.1 | NS | 39.6 | Two | 24 | 2 | 12 |
| Ibrahim, 2018 | 2007-2012 | 72.5 | 46 | >60 | Two | 100 | 6 | 15 |
| Kang, 2018 | 1996-2015 | 66.5 | NS | 88.8 | Two | 85 | 1 | 11 |
| Vcelak, 2018 | 2000-2015 | 61.2 | 59.9 | >24 | Two | 57 | 6 | 12 |
| Wang, 2018 | 2003-2016 | 65 | 50 | 68.5 | Two | 61 | 4 | 13 |
| Akgun, 2019 | 2013-2015 | 70 | NS | 33.1 | Two | 93 | 9 | 15 |
| Ascione, 2019 | 2009-2016 | 66 | 46 | >24 | Two | 120 | 16 | 15 |
| Emfremov, 2019 | NS | 74.8 | 56 | >24 | Two | 30 | 5 | 13 |
| Garcia-Oltra, 2019 | 2002-2010 | 70.4 | 52.2 | 57.2 | Two | 57 | 4 | 11 |
| Jones, 2019 | 2004-2014 | 64 | 58 | 78 | Two | 185 | 20 | 12 |
| Karczweski, 2019 | 2009-2017 | 67 | 43.6 | >24 | Two | 89 | 8 | 13 |
| Matar, 2019 | NS | 63 | 55.2 | 60 | Two | 29 | 1 | 12 |
| Mcalister, 2019 | 2000-2014 | 65.2 | 55 | 60 | Two | 515 | 49 | 13 |
| Petis, 2019 | 1991-2006 | 68 | 65 | 144 | Two | 162 | 23 | 15 |
| Sigmeund, 2019 | 2006-2014 | 76 | 48.4 | 42.7 | Two | 93 | 9 | 11 |
| Svensson, 2019 | 1979-2015 | 68 | 60.3 | 94.8 | Two | 1250 | 107 | 12 |
| Winkler, 2019 | 2013-2013 | 68.4 | 36.8 | 39.5 | Two | 18 | 0 | 16 |
| Zagra, 2019 | 2013-2015 | 64.4 | 55.6 | 32.4 | Two | 54 | 4 | 13 |
| Barton, 2020 | 2008-2015 | 64 | 56.2 | 56.3 | Two | 27 | 2 | 13 |
| Carrega, 2020 | 2010-2012 | 68 | 49 | >24 | Two | 55 | 3 | 12 |
| Crawford, 2020 | 2003-2016 | 66 | 43.5 | 85.2 | Two | 53 | 11 | 12 |
| Sotiriou, 2020 | 2002-2016 | 68 | NS | >24 | Two | 46 | 0 | 12 |
| Theil, 2020 | 2012-2016 | 70 | 49 | 42 | Two | 25 | 93 | 13 |
| Wichern, 2020 | NS | 65.3 | 57.8 | 67 | Two | 54 | 2 | 12 |
| Yang, 2020 | NS | 63.1 | 61.3 | >24 | Two | 63 | 12 | 16 |

Table B.2: Summary characteristics for knee revision arthroplasty;
Study characteristics for knee revision arthroplasty are displayed using the number of patients, the number of reinfections, the mean age in years, mean follow-up in months, and the percentage of males in each study. N: number of patients F/U: follow-up; NS: not specified;

| Study | Study Period | Age in years | %Male | | F/U in months | Stage | N | | Reinfection | Minors |
| --- | --- | --- | --- | --- | --- | --- | --- | --- | --- | --- |
| Jenny, 2016 | NS | 70 | 48.1 | 38 | | One | 131 | 25 | | 14 |
| Massin, 2016 | 2005-2010 | 71 | 49 | 44 | | One | 108 | 25 | | 11 |
| Leta, 2019 | 1994-2016 | 67 | 37.5 | 61.2 | | One | 72 | 10 | | 14 |
| Siddiqi, 2019 | 2012-2017 | NS | 52.6 | 52.9 | | One | 57 | 8 | | 13 |
| Ji, 2020 | 2009-2016 | 62.7 | 43.2 | >24 | | One | 139 | 10 | | 13 |
| Pellegrini, 2020 | 2009-2016 | 67.6 | 45 | 74.4 | | One | 20 | 0 | | 14 |
| Berend, 2015 | 2005-2013 | 64 | 48 | 36 | | Two | 182 | 28 | | 12 |
| Cha, 2015 | 1998-2011 | 66.5 | 25 | 30.3 | | Two | 88 | 18 | | 12 |
| Sakkellariou, 2015 | 2000-2011 | 64.3 | 46.7 | >24 | | Two | 110 | 15 | | 15 |
| Wilke, 2015 | 1994-2002 | 67 | 45.3 | 90 | | Two | 75 | 19 | | 13 |
| Chen, 2016 | 1999-2012 | 71.1 | 22.2 | >24 | | Two | 19 | 4 | | 12 |
| Drexler, 2016 | 2006-2012 | 68 | 52.4 | 36 | | Two | 93 | 12 | | 16 |
| Hoell, 2016 | 2004-2008 | 73 | 54.2 | 49.2 | | Two | 59 | 16 | | 12 |
| Juul, 2016 | 2010-2013 | 65.7 | 45.5 | 37.6 | | Two | 22 | 4 | | 11 |
| Lackey, 2016 | 2005-2013 | NS | NS | >24 | | Two | 74 | 6 | | 13 |
| Lindberg-Larsen, 2016 | 2011-2013 | 69 | 60 | 38.4 | | Two | 205 | 50 | | 14 |
| Marczak, 2016 | 2008-2012 | 68.5 | 28.6 | 52 | | Two | 56 | 5 | | 14 |
| Massin, 2016 | 2005-2010 | 67 | 45 | 55 | | Two | 177 | 55 | | 11 |
| Vasso, 2016 | 2000-2010 | 69 | 30.4 | 144 | | Two | 46 | 2 | | 13 |
| Buyuk, 2017 | 2005-2012 | 70 | 16 | 39.8 | | Two | 31 | 0 | | 12 |
| Triantafyllopoulos, 2017 | 1998-2014 | 65.4 | 51.6 | 132.2 | | Two | 266 | 24 | | 13 |
| Dwyer, 2018 | 2000-2014 | 63.4 | 51.7 | 62.4 | | Two | 132 | 39 | | 12 |
| Ford, 2018 | 2004-2014 | 65.4 | NS | 39.6 | | Two | 56 | 16 | | 12 |
| Henry, 2018 | 2009-2014 | 66 | 63 | >24 | | Two | 159 | 24 | | 12 |
| Ma, 2018 | 2005-2015 | 70.5 | 27.4 | 67.2 | | Two | 106 | 16 | | 11 |
| Perry, 2018 | 2000-2012 | 64 | 59 | 78 | | Two | 54 | 3 | | 11 |
| Akhtar, 2019 | 2009-2015 | 75.2 | 52.2 | 44.2 | | Two | 44 | 4 | | 10 |
| Ascione, 2019 | 2009-2016 | 66 | 46 | >24 | | Two | 92 | 11 | | 15 |
| Chang, 2019 | 2003-2013 | 73.3 | 46.6 | 46.2 | | Two | 58 | 13 | | 11 |
| Emfremov, 2019 | NS | 71.2 | 53 | >24 | | Two | 32 | 4 | | 13 |
| Garceau, 2019 | 2001-2018 | 67.9 | 53.3 | 75 | | Two | 137 | 28 | | 15 |
| Grzelecki, 2019 | 2007-2015 | 67.9 | 23.5 | 53.3 | | Two | 143 | 38 | | 15 |
| Karczweski, 2019 | 2009-2017 | 68.3 | 43.6 | >24 | | Two | 86 | 19 | | 13 |
| Leta, 2019 | 1994-2016 | 69 | 43.2 | 61.2 | | Two | 243 | 28 | | 14 |
| Ma, 2019 | 2003-2009 | 70.3 | 32.8 | 75.3 | | Two | 66 | 14 | | 14 |
| Petis, 2019 | 1991-2006 | 68 | 50 | 168 | | Two | 245 | 42 | | 15 |
| Siddiqi, 2019 | 2012-2017 | NS | 35.8 | 54.7 | | Two | 137 | 33 | | 13 |
| Sukontahong, 2019 | 2003-2013 | 66.7 | 41.9 | >24 | | Two | 40 | 4 | | 12 |
| Tsai, 2019 | 2015-2015 | 73.3 | 43.8 | 36.9 | | Two | 32 | 4 | | 13 |
| Winkler, 2019 | 2013-2013 | 68.4 | 36.8 | 39.5 | | Two | 20 | 0 | | 16 |
| Barton, 2020 | 2008-2015 | 64 | 56.2 | 56.3 | | Two | 62 | 7 | | 13 |
| Barry, 2020 | 2005-2016 | 66.3 | 45.1 | 42 | | Two | 31 | 10 | | 11 |
| Bongers, 2020 | 2003-2013 | 67 | 45 | 94 | | Two | 113 | 23 | | 15 |
| Caglar, 2020 | 1999-2009 | 61 | 28.1 | 84 | | Two | 42 | 2 | | 12 |
| Carrega, 2020 | 2010-2012 | 68 | 49 | >24 | | Two | 47 | 3 | | 12 |
| Chen, 2020 | 2003-2013 | 69.5 | 51.7 | 65.1 | | Two | 58 | 13 | | 13 |
| Faschingbauer, 2020 | 2002-2010 | 69.9 | 53.1 | >24 | | Two | 137 | 18 | | 13 |
| Gogelioglu, 2020 | 2011-2017 | 67 | 37.5 | 48 | | Two | 56 | 10 | | 12 |
| Kim, 2020 | 2011-2016 | 69 | 17.1 | 32.9 | | Two | 70 | 17 | | 11 |
| Ma, 2020 | 2003-2009 | 70.3 | 32.8 | 75.3 | | Two | 66 | 14 | | 12 |
| Nahhas, 2020 | 2011-2016 | 65.3 | 46.9 | 42 | | Two | 68 | 3 | | 15 |
| Seo, 2020 | 2009-2013 | 70.7 | 59.6 | >36 | | Two | 14 | 0 | | 14 |
| Theil, 2020 | 2012-2016 | 70 | 49 | 42 | | Two | 111 | 44 | | 13 |
| Yang, 2020 | NS | 63.1 | 61.3 | >24 | | Two | 79 | 17 | | 16 |
| Zamora, 2020 | 2012-2017 | 69 | 49 | >24 | | Two | 47 | 8 | | 12 |

**Appendix C. Subgroup Analysis and Meta regression:**

A p-value <0.05 was considered significant

**One-stage hip revision**

By region:


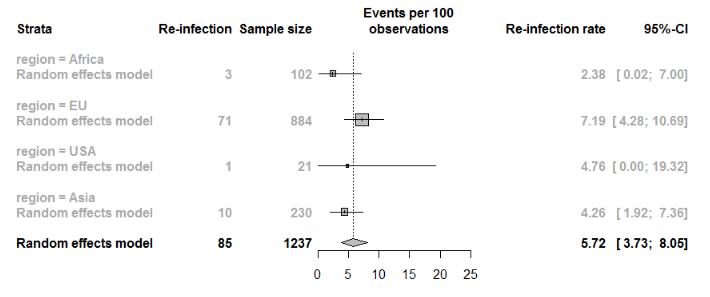


Meta-regression P-value = 0.49

Figure C.1 Re-infection rates of one-stage hip revision grouped by region

By age:


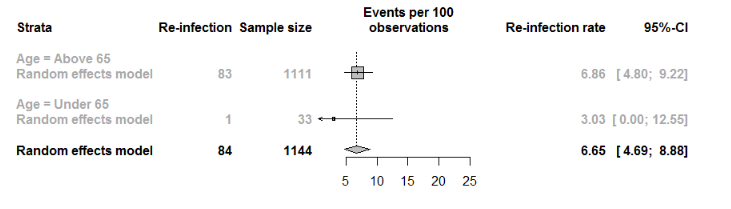


Meta-regression P-value = 0.45

Figure C.2 Re-infection rates of one-stage hip revision grouped by age (different values for re-infection and sample size due to missing data)

By study size:


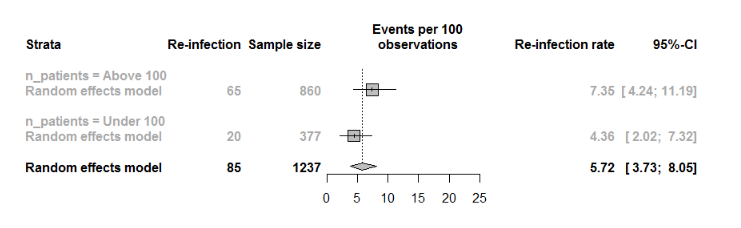


Meta-regression P-value = 0.35

Figure C.3 Re-infection rates of one-stage hip revision grouped by study size

By MINORS score:


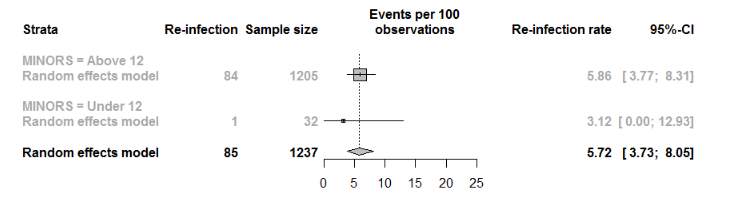


Meta-regression P-value = 0.63

Figure C.4 Re-infection rates of one-stage hip revision grouped by MINORS-score

**Two-stage hip revision**

By region:


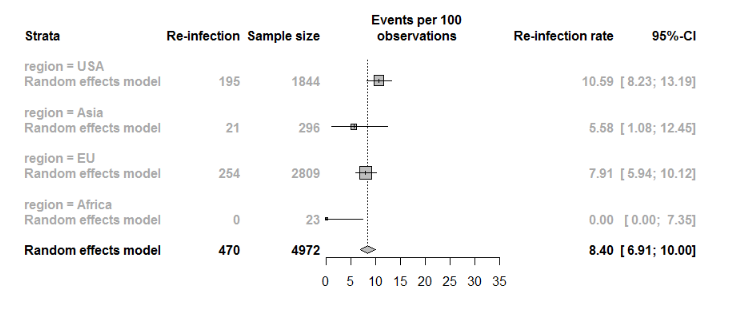


Meta-regression P-value = 0.09

Figure C.5 Re-infection rates of two-stage hip revision grouped by region (different values for re-infection and sample size due to missing data)

By age:


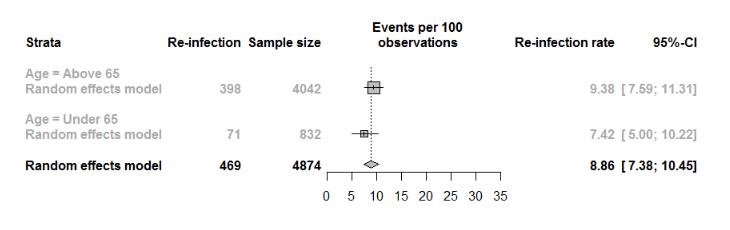


Meta-regression P-value = 0.45

Figure C.6 Re-infection rates of two-stage hip revision grouped by age (different values for re-infection and sample size due to missing data)

By study size:


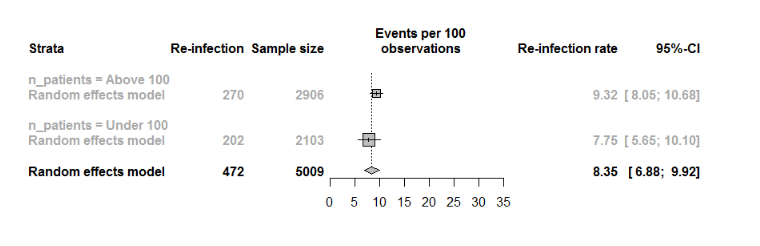


Meta-regression P-value = 0.53

Figure C.7 Re-infection rates of two-stage hip revision grouped by study size

By MINORS score:


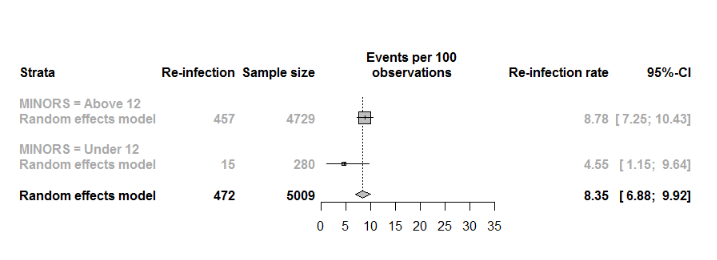


Meta-regression P-value = 0.07

Figure C.8 Re-infection rates of two-stage hip revision grouped by MINORS score

By publication year:


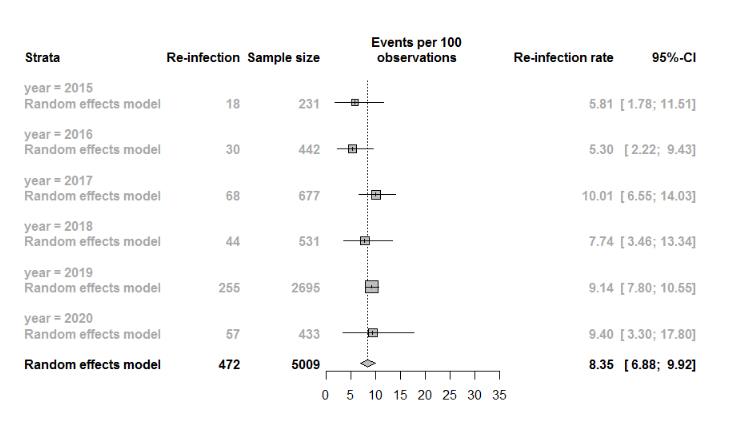


Meta-regression P-value = 0.10

Figure C.9 Re-infection rates of two-stage hip revision grouped by publication year

**One-stage knee revision**

By region:


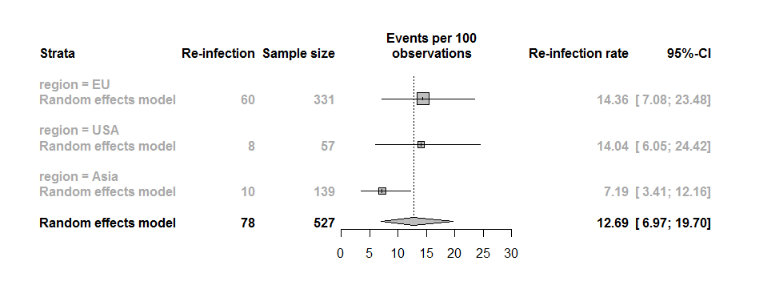


Meta-regression P-value = 0.59

Figure C.10 Re-infection rates of two-stage knee revision grouped by region

By age:


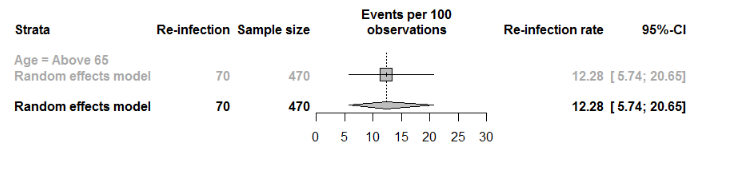


Only 1-strata

Figure C.11 Re-infection rates of two-stage hip revision grouped by age (different values for re-infection and sample size due to missing data)

By study size:


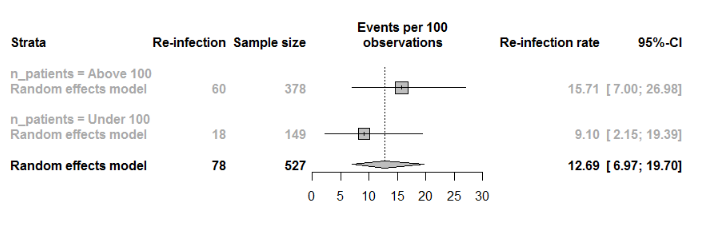


Meta-regression P-value = 0.39

Figure C.12 Re-infection rates of two-stage hip revision grouped by study size

By MINORS score


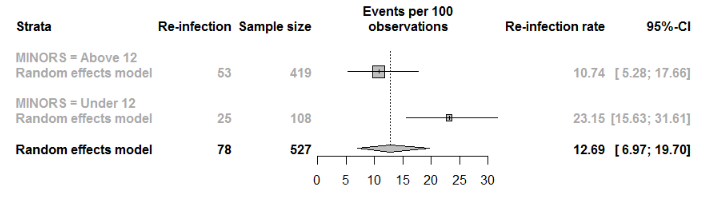


Meta-regression P-value = 0.16

Figure C.13 Re-infection rates of two-stage hip revision grouped by MINORS score

**Two-stage knee revision**

By region:


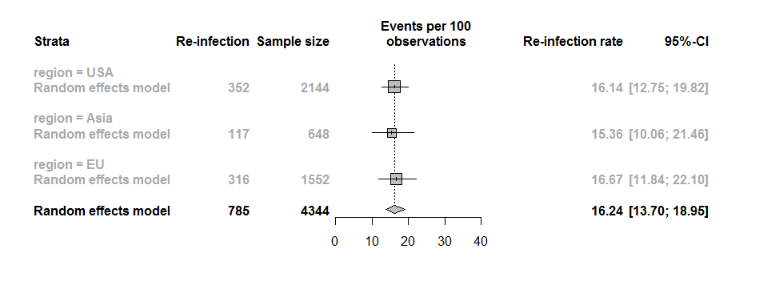


Meta-regression P-value = 0.93

Figure C.14 Re-infection rates of two-stage hip revision grouped by region

By age:


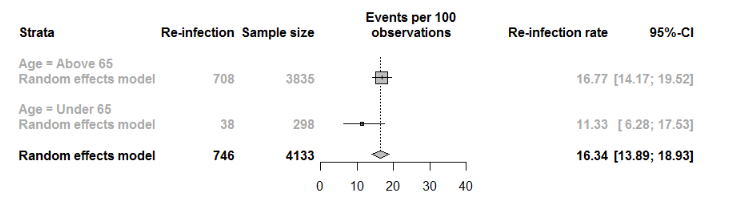


Meta-regression P-value = 0.21

Figure C.15 Re-infection rates of two-stage hip revision grouped by age (different values for re-infection and sample size due to missing data)

By study size:


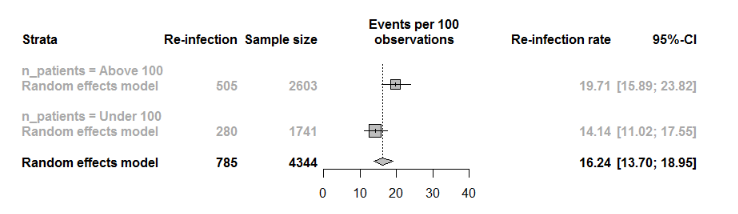


Meta-regression P-value = 0.06

Figure C.16 Re-infection rates of two-stage hip revision grouped by study size

By MINORS score:


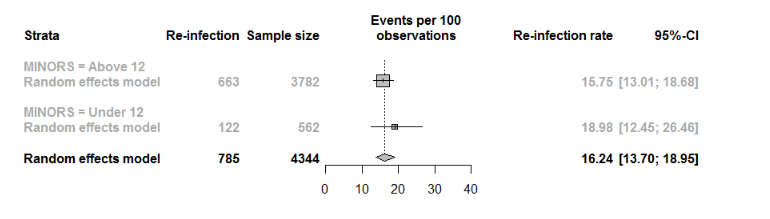


Meta-regression P-value = 0.36

Figure C.17 Re-infection rates of two-stage hip revision grouped by MINORS score

By publication year


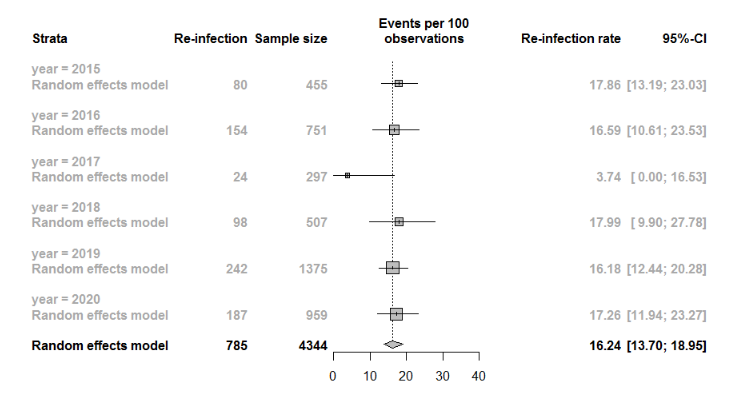


Meta-regression P-value = 0.91

Figure C.18 Re-infection rates of two-stage hip revision grouped by publication year
